# Overview of Polio Outbreak Response in Kenya, 2013 to 2015

**DOI:** 10.29245/2578-3009/2021/S2.1103

**Published:** 2021-04-02

**Authors:** Chidiadi Nwogu, Johnny Musyoka, Carolyne Gathenji, Rosemary Nzunza, Iheoma Onuekwusi, Joseph Okeibunor, Pascal Mkanda, Hemant Shukla, Shaikh Humayun Kabir, Sam O Okiror

**Affiliations:** 1 WHO Horn of Africa Coordination Office (HOA), Nairobi Kenya; 2 Ministry of Health (MoH), Nairobi, Kenya; 3 WHO, Nairobi, Kenya; 4 Kenya Medical Research Institute (KEMRI), Nairobi, Kenya; 5 WHO Regional Office for Africa, Brazzaville, Congo

## Abstract

**Background:**

Globally, tremendous improvement has been made in Polio eradication since its inception in 1988. For the third time in a decade, Kenya has experienced a Polio outbreak along the border with Somalia. The affected areas were in Garissa County, replete with previous occurrences in 2006 and 2012. This article, give an account of series of events and activities that were used to stop the transmission within 13 weeks, an interval between the first and the last case of the 2013 outbreak.

**Methods:**

In an attempt to stop further transmission and time bound closure of the outbreak, many activities were brought to fore: the known traditional methods, innovative approaches, improved finances and surge capacity. These assisted in case detection, implementation, and coordination of activities. The external outbreak assessments and the six-monthly technical advisory group recommendations were also employed.

**Result:**

There were increased case detections of >=2/100,000, stool adequacy >=80%, due to enhanced surveillance, timely feedbacks from laboratory investigation and diagnosis. Sustained coverage in supplemental immunisation of > 90%, ensured that immune profile of >=3 polio vaccine doses was quickly attained to protect the targeted population, prevent further polio infection and eventual reduction of cases coming up with paralysis.

**Conclusion:**

Overall, the outbreak was stopped within the 120 days of the first case using 14 rounds of supplemental immunisation activities.

## Introduction

Globally, tremendous improvement has been made in polio eradication since its inception in 1988^1^. However, the erratic occurrence of wild poliovirus type 1 (WPV1) outbreaks in certain countries of Africa and South-East Asia have always set the hands of the clock back, each time^2^. These countries have few things in common: issues of conflict, insecurity, and inaccessibility.

As of 2013, Kenya, a country in the Horn of Africa had an estimated population of 43 million^3^. Kenya is bordered to the North by Ethiopia, northeast by Somalia, South East by the Indian Ocean, southwest by Tanzania and North West by Uganda and South Sudan. Kenya’s proximity to polio outbreak-prone countries increases its vulnerability to the importation of wild poliovirus.

Kenya experienced two wild poliovirus outbreaks in 2006 in the then Garissa District (now a County) with dates of onset of paralysis on 17 September and 13 November respectively. In February 2009, a wild poliovirus importation from South Sudan resulted in 19 cases in Turkana County. The July 2011 WPV outbreak detected in Kamagambo, Rongo district of Kenya was an importation from Uganda and was genetically linked to the 2009 outbreak in Turkana, Kenya and the 2010 outbreak in Bugiri districts of Uganda. In July 2012, the Dadaab refugee camps recorded two cases of circulating vaccine-derived poliovirus. Response to each outbreak was according to WHO guidelines which required immediate outbreak investigation, vaccination response around the cases, strengthening of AFP surveillance and enhancing routine immunisation^4^. Again, between May and July 2013, 14 WPV 1 cases occurred along the axis of Dadaab, Fafi and Hulugho sub-Counties of Garissa County in Kenya, all linked to Somalia. One element common to these outbreaks was proximity to the border areas between Kenya and its neighbouring countries. Moreover, the 2013 outbreak had a shift to the above 15 years age groups contrary to what has been experienced before in Kenya. However, this is not unique to Kenya as it has occurred in Namibia and the Democratic Republic of, Congo. This is epidemiologically important in that the intervention will no longer be restricted to children aged <5 years but also to the older age groups^5^.

The 2013 outbreak was declared closed in June 2015, and this article gives an account of the series of events and activities that were used to stop the transmission within 90 days of the onset of the first case. This was in line with the within 120 days period to stop the transmission as recommended by WHO^6^.

The article also, documents for the first time the activities that were undertaken in response to the 2013 polio outbreak in Kenya for future references.

## Methods

### Traditional and innovative approaches used

In Garissa County, we used enhanced surveillance activities which raised the non-polio acute flaccid paralysis (NP-AFP) indicator from the ≥2/100,000 of the under 15 years to ≥3/100,000 aimed at increasing case detection^6^. This became possible with the improved finances that increased the number of districts field staffers recruited to detect cases through active case search and desk reviews from the communities and facilities. All cases detected were promptly investigated by the District surveillance officers. Moreover, the Kenyan program established permanent and transit vaccination points to vaccinate children moving in and out of the border areas between Kenya and South Sudan.

### Advocacy communication and social mobilization (ACSM) activities

Health workers were trained on interpersonal communication skills, and polio ambassadors were selected and trained to reach out to the resistant communities. Advocacy, communication and social mobilisation committees were formed at the county and sub-county levels to monitor social mobilisation activities. Besides, funds were made available for communication for development activities and social mapping of mobile populations and tracking the nomadic pastoralists.

### Supplemental Immunization Activities (SIAs)

These include national immunisation days (NIDs) implemented in all 47 Counties of Kenya and subnational immunisation days (SNIDs) in selected high-risk Counties. Short interval additional doses (SIADs) given at 2 weeks interval were also used to attain high protection levels in high-risk areas quickly. Extended age group vaccination considered the epidemiological trend of the outbreak was shifting to the older age groups through the vaccination for all the age groups in Garissa County, the epicentre of the outbreak. The introduction of inactivated polio vaccine targeting 6 to 59 months age group in the refugee camps and surrounding host communities was in line with the Global end game strategy.

### Laboratory functions

Detected cases had their stool samples sent to the laboratory to identify those positive for either WPV or circulating vaccine derived polioviruses cVDPVs^7^. Many stool specimens from the AFP cases detected, and contact stools increased the workload at the Laboratory. This was overcome by procurement of laboratory supplies, temporary recall of retired staff and recruitment to narrow surge capacity. Equipment that could not be accessed were borrowed from affiliate Partners.

### Mapping of mobile populations

Mapping of mobile populations in Turkana and Garissa Counties by the community elders and government functionaries using a tracking format to update new settlements in the micro-plans. The update directed the assignment of teams and materials in subsequent SIA rounds.

### Staff needs assessment

Staff needs assessment conducted by WHO country office in Kenya, revealed a capacity gap for the outbreak response. Given this, consultants were recruited and deployed at several times to low performing areas for technical support. A subsequent review of the surge capacity found it necessary to recruit four additional incountry staff to reinforce the surge. The arrival of the CDC Stop Transmission of Polio (STOP) teams, were also deployed to the affected areas to assist in the outbreak response implementation.

### External assessment of outbreak response and Technical Advisory Group (TAG) meetings

An external inter-agency team assessed the outbreak responses as per WHO standard operating procedure (SOP). As per World Health Assembly (WHA) resolution, 2006, the updated assessment strategies of WHO recommends, to conduct external outbreak assessments at 3-month intervals, until 6 months have passed without WPV/ cVDPV identification and if after 6 months there is none, the Country should conduct a final external assessment. The objectives of these assessments were first to assess the quality and adequacy of polio outbreak response activities, and second is to evaluate if the response is on track to interrupt polio transmission within six months of first case detection and provide additional technical recommendations to assist the country in meeting the goal of interrupting transmission. The assessments focused on many areas of finding: if the speed and appropriateness of immediate outbreak response were in line with the resolution, the effectiveness of government leadership and that of the partners’ coordinating the outbreak response. Others were: the quality of SIAs concerning planning, delivery, monitoring and communication, quality of AFP surveillance activities, routine immunisation performance and adequacy of human resource for the response. The findings had varied between the assessments. Also, recommendations from the six- monthly TAG meetings were also implemented.

## Study Population

The study population varied for different response strategies: for surveillance, we focused on children below 15 years and for supplemental immunisation activities (SIAs), 0 days to 59 months. The extended vaccination event was for all ages, and the introduction of the inactivated polio vaccine (IPV) covered children aged between 6 to 59 months. The trend analysis of the immune profile status was for the 6 to 59 months non-polio acute flaccid paralysis (NPAFP) cases for the period, 2012 to June 2015.

## Study Location

For surveillance, we conducted a trend analysis for immune profile and national immunisation days for all the 47 counties of Kenya. The sub-National immunisation days were in the selected high-risk areas of Kenya and areas (refugee Camps and host Community) with IPV introduction.

## Data Collection

We collected data from activities carried out during the outbreak response in Kenya. These were: multiple crosssectional data for surveillance response, post-evaluation of SIA rounds using independent monitoring (IM) for SIA response.

## Data Analysis

We conducted trend analyses for missed children during 2nd to 4th quarter 2014 SIA rounds preceding the closure of the outbreak and immune status of NPAFPs 6 to 59 months age group from 2012 to June 2015. Other data sources were from external outbreak assessment and recommendations of the horn of Africa (HOA) TAG meeting.

## Results

From 2012 to 2015, AFP case detection based on the NP-AFP rate was above the minimum of 2/100,000 population of children <15 years old. Within the same period, specimen arriving laboratory in good condition was >90% except in 2013 it was 74%. Other surveillance indicators like: stool adequacy rate, cases investigated within 2 days of notification, specimen arriving laboratory within 3 days since collection and laboratory result at program within 14 days of receipt were >80%. However, non-polio enterovirus isolation was above 10% in 2012 and 2013 but reduced in 2014 and 2015 (Table 1).

In 2013 outbreak, 14 wild polioviruses type one confirmed in Kenya laboratory were all from Garissa County and the time interval between the first and the last case was 13 weeks (Table 2). The increase in the Laboratory workload had impacted on timely result generation, stressed routine procedures, compromised supervisory processes and created unprecedented programmatic contamination and misreporting.

**Figure 1:**
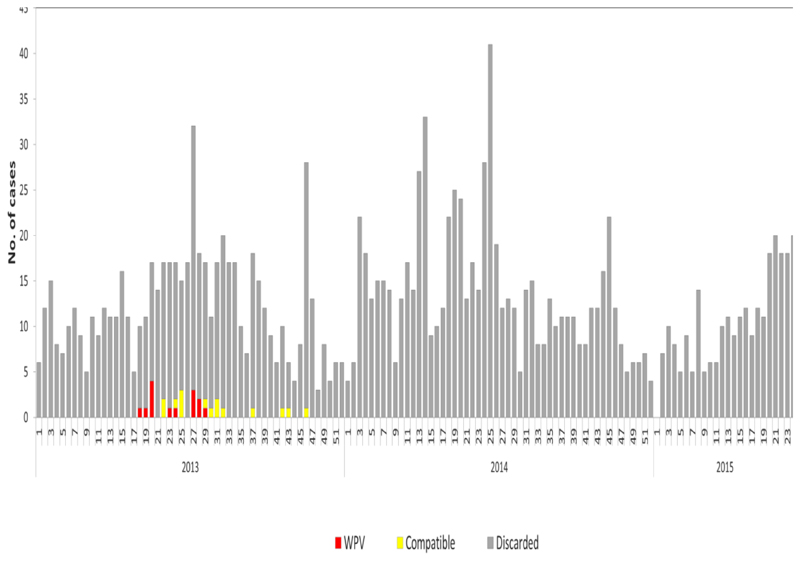
Epi Curve showing AFP by week of onset, Wild polio-virus outbreaks, and supplemental immunization activities (SIAs), Kenya 2013 -2015 (wk24).

**Figure 2:**
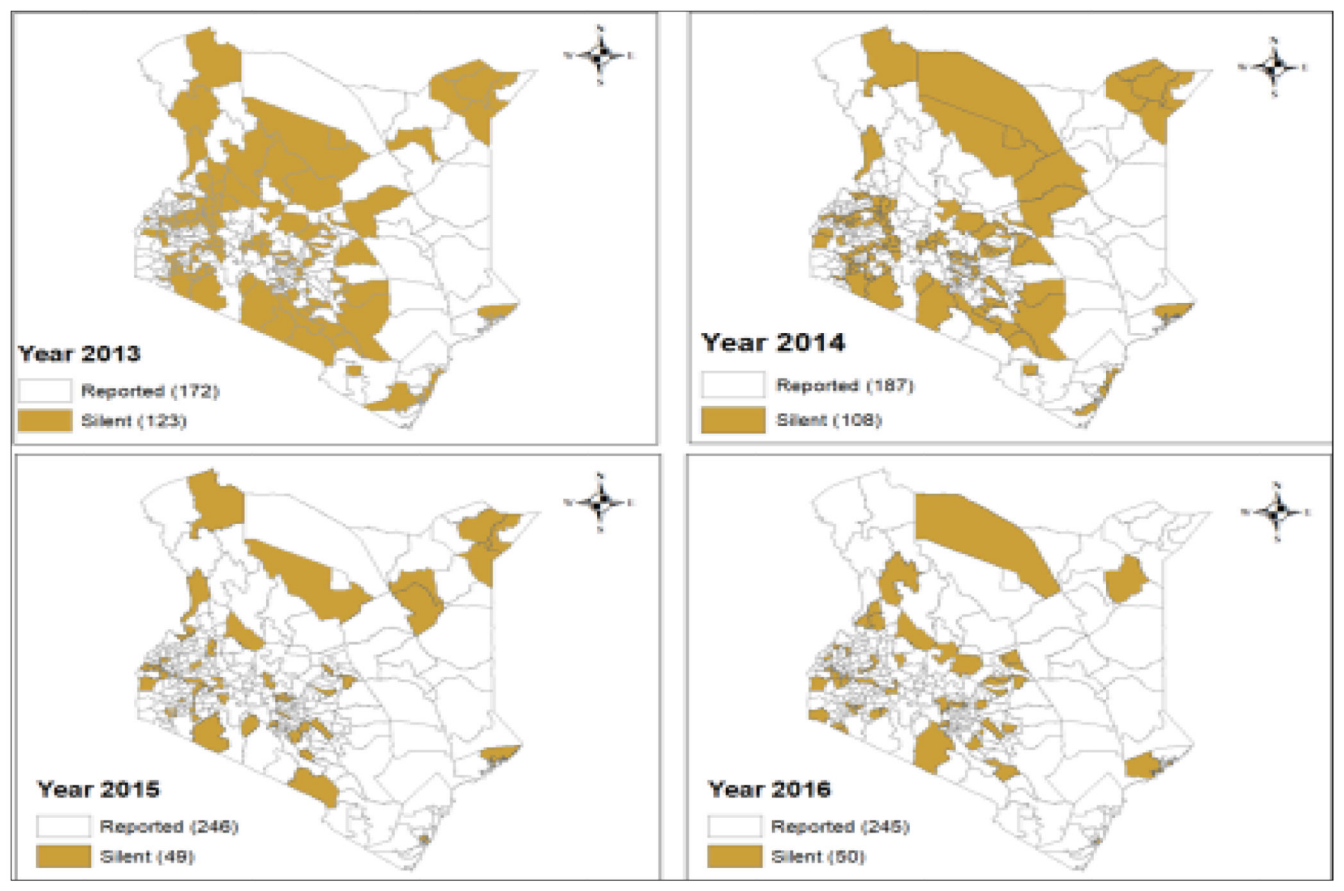
Map of Silent Districts in Kenya 2013 – 2016 showing reduction in number of silent districts in view of improved surveillance activities during the period (Source, WHO, Kenya Database, 2016)

The epidemiologic curve shows the week of the outbreaks in 2013, AFP cases detected and classified, and the eventual 14 rounds of SIAs employed. It started as an importation from Somalia with one case having a date of onset of 30/04/2013 within epidemiologic week 18, at the subsequent week another case was confirmed. Efforts to contain the virus and eventually stop transmission were: three high-quality polio SIAs carried out between week 22 and week 27 bringing the outbreak to a stop in 13 weeks. Within the 13 weeks of active WPV1 transmission, 14 cases were reported, with the highest number of cases being on the third week of transmission at week 20. Implementation of high-quality SIAs had continued at National and subnational levels to a total of 14 rounds by the end of 2014 to ensure high population immunity. There was also the emergence of polio compatibles during and after stopping transmission, signifying staff sincerity in reporting and suboptimal surveillance activity necessitating heightening of surveillance measures. The last compatible case was reported during epidemiologic-week 46 of 2013 (Figure 1). Due to intensive surveillance activities during the response, the number of silent districts out of the reporting districts by year reduced from 71.5% (123/172) in 2013 to 20.4% (50/245) in 2016 (Figure 2).

**Figure 3:**
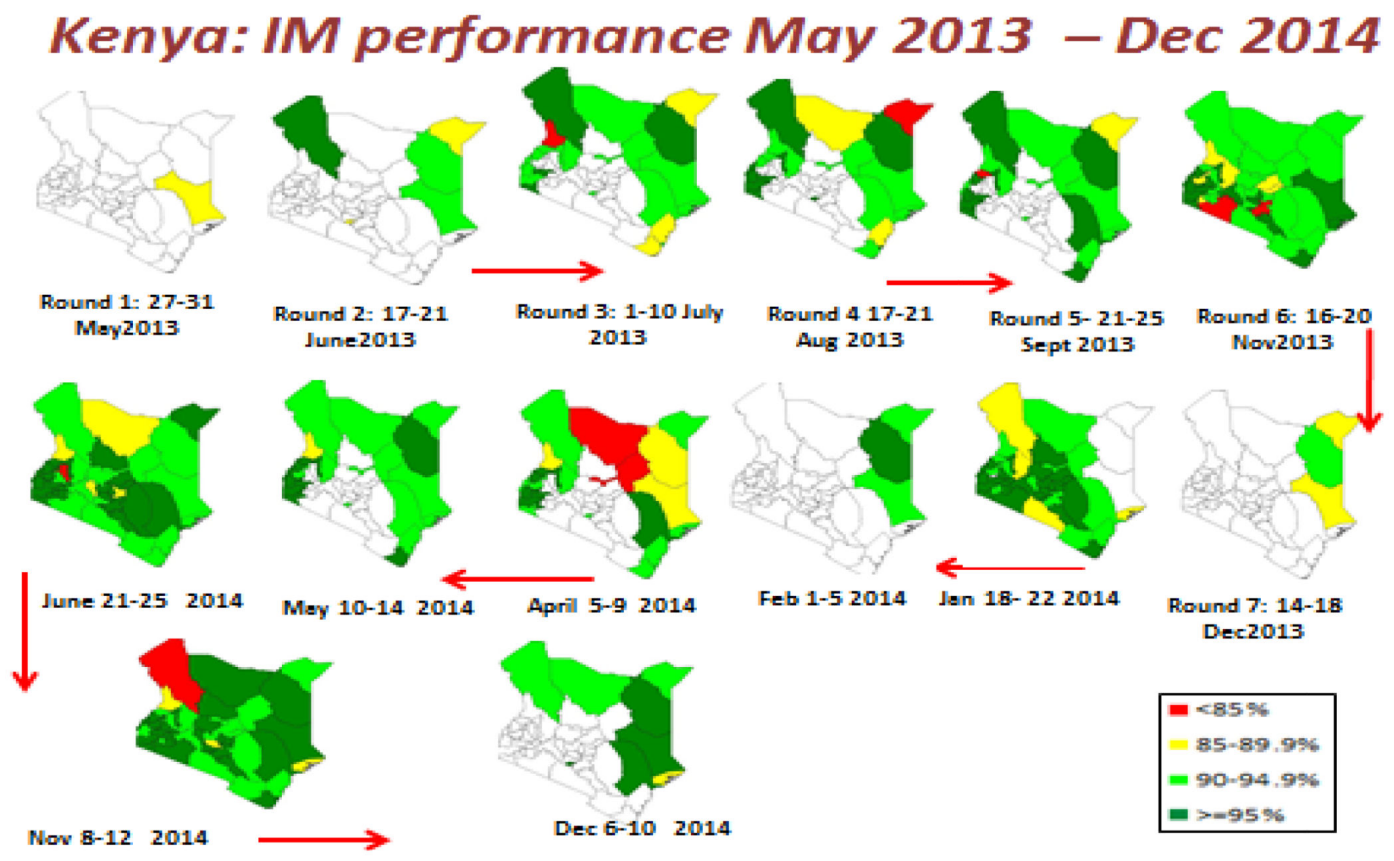
Showing number of Supplemental immunization activity (SIA) rounds and IM Outcome, Kenya May 2013 to December 2014. (Source, Final HOA Outbreak Assessment for Kenya, June 2015)^24^

During the NID in June 2014, more than 41 (85%) of the 47 Counties had independent monitoring IM coverage of ≥90%. Campaign awareness increased from 89% in November to 92% in December 2014 and was followed by a commensurate reduction in missed children to below 2% in 8 (89%) out of the 9 priority Counties within the same period. Even though there was this reduction, Lamu County continued to have missed children of more than 5% while Turkana had missed children increased in December 2014 round (Figure 3).

**Figure 4:**
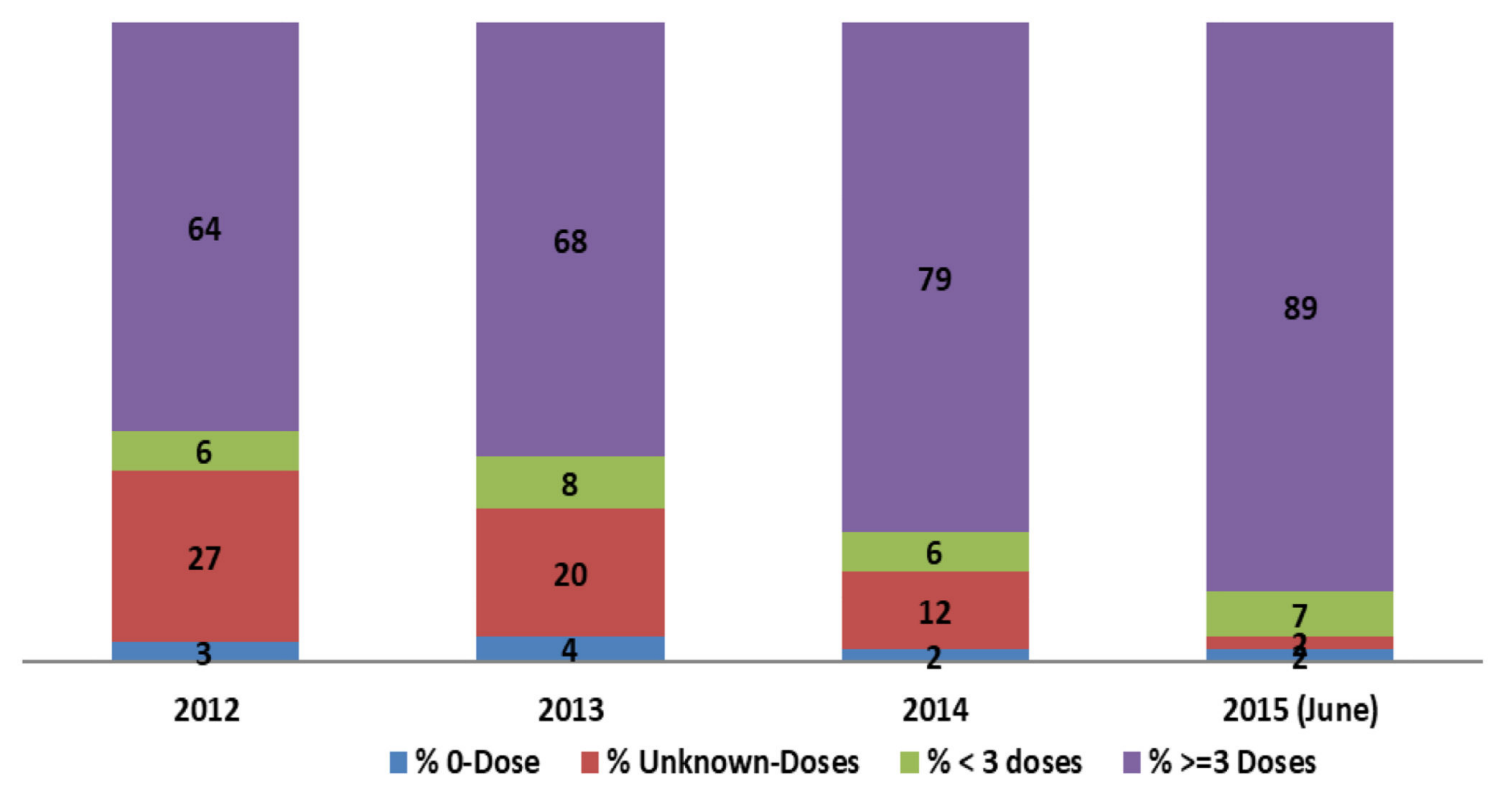
Shows Immune Profile of NPAFPs (6 -59months), Kenya 2012- 2015 (June)^26^ (Source: 2015 final Outbreak response Assessment, Kenya)^24^

Between 2012 and 2015, there was an increase in the protection capacity immune status of the non-polio AFP cases receiving three and above doses of oral polio vaccine and a commensurate decrease in cases with below three, unknown and zero doses (Figure 4).

**Figure 5:**
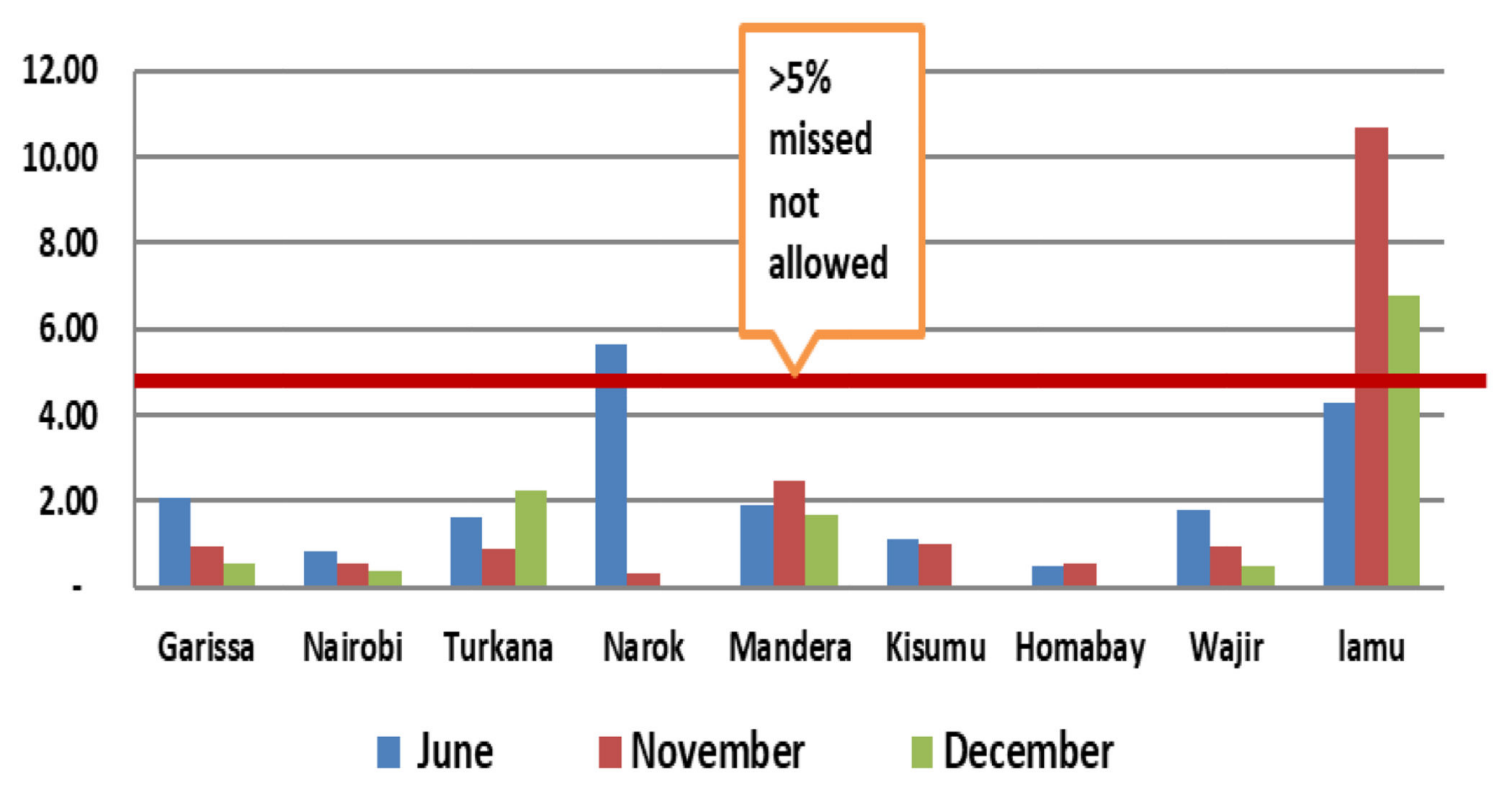
Reduction in % Missed children in 8 of 9 Counties in Kenya SIA rounds 2014 (Source, Independent Monitoring, and Kenya 2014)

Within the last three rounds of 2014, there was a reduction in missed children in 8 out of the 9 high-risk Counties except for Turkana. Even though there was this reduction, Lamu County continued to have missed children more than 5% (Figure 5).

## Assessments Findings

Kenya responded on time and through a dedicated public health staff and strong government leadership with efficient partner’s coordination. The quality of the response activities is unlikely to achieve sufficient population protection (immunity) required to interrupt WPV transmission within 6 months of index case detection. The quality of SIAs in the high-risk areas except Dadaab refugee Camps was suboptimal, and there were gaps in AFP surveillance at sub national levels which may prevent early detection and response. The team concluded that the risk of WPV importations remains high and to minimise the risk of further spread of WPVs, population immunity must be at the highest level.

At the second assessment, the team noted: excellent tracking of 0-weekly reports, regular feedbacks to the communities and piloting of community-based surveillance in three high-risk Counties. From the laboratory, there was an improvement in the environmental sampling established at six high-risk sites, handling of laboratory responsibilities and the contact sampling of inadequate AFP cases. In communication, social profiling of WPV/ VDPV cases and integration of social data into the micro plans, real time monitoring through mobile platforms and engagement of religious and clan leaders. The team noted the functioning of the “Mtoto Kwa Mtoto” - a school strategy used by children to identify missed children and referring them for vaccination through their parents.

## Discussion

We found that, throughout the period (pre and outbreak), the NP-AFP and percent stool adequacy remained above the minimum recommended by the World Health Organization (WHO). However, the sharp drop in NP-AFP rate from 3.8 in 2014 to 2.8 per 100,000 in the first quarter of 2015 necessitated that in May 2015, a national surveillance review was conducted. After this review, several gaps to effective AFP surveillance were identified^7,8^. These were: lack of trained surveillance teams, inadequate and irregular funding, and sub-optimal supervision particularly from the National level, lack of advocacy for surveillance at management level and insufficient engagement of existing community units to strengthen surveillance. Consequently, a surveillance enhancement plan was developed and implemented. The improvement in surveillance indicators after was a testimony to the impact of the review.

In the continuum of AFP surveillance, the Global Polio Laboratory Network (GPLN) procedures are key^9^. The first confirmed case with onset of paralysis on 30th April 2013 came from an AFP contact in Dadaab refugee camp whom stool samples were collected on 5th and received on 9^th^ of May 2013 at Kenya Medical Research Institute (KEMRI). The laboratory response following this outcome resulted in the collection of 3,520 stool samples from AFP cases, their contacts and healthy individuals. Healthy children stool samples collection in areas that have remained silent for reporting AFP cases >8 weeks is crucial for detecting low-level transmission.

Open defecation in the periphery of most towns and cities is one of the unhealthy lifestyle of the people in the North Eastern Counties of Kenya and in many Countries of Africa where polio infections have persisted. Moreover, open defecation encourages the shedding of poliovirus into the environment. Given the pathogenesis of polio; hand to mouth attitude of children and some adults play a major role in Polio infection. In addition, there are many congested city areas and in some sub Counties with high degree of poor hygiene and seeping sewages. This is with particular reference to Kamukunji area in Nairobi. It is also a common knowledge that the mere absence or presence of obvious clinical symptoms do not stop poliovirus replication which is understood to continue in the para-intestinal sub mucosal lymphatic tissue for several weeks. These, have all been considered in the establishment of environmental surveillance in Kenya, October 2013. The establishment aimed at collecting samples from drainage and sewage systems to compliment surveillance for acute flaccid paralysis (AFP). Between 2012 and 2015, Nigeria made timely use of information from environmental surveillance to trigger public health interventions that contributed to the progress made toward the interruption of poliovirus transmission^10,11^. Experience from Nigeria confirms that ES can detect the introduction and silent circulation of WPV and cVDPV^12^. Policies of embarking on provision of effective Latrines in all these areas, and timely closure of broken and seeping sewages by the County and sub-County administration will curb polio transmission and other communicable diseases infections in the Country.

The essence of prompt intervention in polio outbreaks among others is to boost the immunity status of the vulnerable population through SIAs while also enhancing the routine immunisation. The prospect of combining SIAs, routine immunisation with improved surveillance helps to avoid further spread and aims at stopping transmission using WHO guidelines^13,14^. The immunization response activity needed Fourteen SIA rounds which included, 3 NIDs and11 SNIDs were conducted. Considering the epidemiological shift of the outbreak towards the older age group necessitated that all age group vaccination was employed in Garrisa and Wajir Counties^15^. Also, given the low routine immunisation coverage in the area, required that a pilot introduction of inactivated polio vaccine (IPV) in the refugee Camps and the host community was conducted^16–18^. Moreover, the establishment of permanent and transit vaccination points (P/TVPs) at the Kakuma refugee Camps and the Nadpal border with South Sudan ensured that refugees and migrant children were vaccinated^16^. The boost in the immune status of the targeted due to the increased vaccine doses that were given determines the level of protection acquired, capable of preventing polio infection. The impact was an increase in the immune status of the >=3 and a commensurate decrease in <3, unknown and 0-dose polio vaccines among the NP-AFP cases aged between 6 to 59 months. The outcome was documented after the independent monitoring (IM) and pilot Lots quality assurance survey (LQAS) surveys aimed at evaluating the SIA quality^17^.

Polio communication reviews as seen in India and Pakistan have supported improvement in the collection, analysis, and use of data during polio outbreak responses^18^. Kenya implemented communication strategies which ensured that health workers and supervisors were trained on interpersonal communication (IPC) skills. During the SIAs rounds, funds were provided to improve communication skills in poor performing districts, border points, resistant groups and implementation of the school strategy where pupils and teachers sensitised >30,000 households in Turkana and Garissa Counties. It aided formation and training of County and sub-County committees that supported planning, implementation and monitoring of advocacy communication and social mobilization (ACSM) activities. The strategy further enhanced the Polio ambassador reaching out to the immunisation resistant Churches. In Northern Nigeria, the implementation of these activities during SIAs assisted in breaking community barriers to polio vaccination through grassroots mobilisation^19^. The impact was the immediate increase in campaign awareness, a commensurate reduction in missed children in 8 of the 9 priority Counties and SIAs IM coverage of ≥90%.

The recommendations from the first, second and final assessments were conducted as in the guidelines^20^. The final assessment in June 2015 concluded that the government authorities and support partners had played their roles perfectly and outbreak response has met the global standard. The team also noted that previous recommendations had been implemented to an outstanding extent and there was ground evidence suggesting that Kenya has interrupted transmission. The ground evidence were a significant improvement in the population immunity, increased surveillance sensitivity to detect all transmission even though some sub-National gaps exist, excellent communication strategy put in place though needing updating and strengthening, and high campaigns acceptance despite repeated SIAs. Given these conclusions, Kenya was adjudged to have interrupted transmission requiring the closure of the outbreak with recommendations^21^. These outcomes were also reinforced by the Technical Advisory Group (TAG) meetings for HOA in Kenya.

Effective coordination has contributed to the success of closing the outbreak. Before this outbreak, the African regional conference on immunisation in 2010, noted that “The possibility of interrupting transmission in Countries without effective coordination is always difficult”^22^. The Kenya Ministry of Health initially coordinated the outbreak response with few partners, from the National level to the facility catchments at the community level.

However, the establishment of the HOA coordination group enabled a unified approach towards supporting Kenya and other HOA countries through the production of technical updates and Bulletins. These documents shared information on the situation analysis, epidemiology, planned SIAs and IM coverage data, and resource requirements. The Horn of Africa (HOA) coordination group was born out of the Independent monitoring board (IMB) recommendation, at its 8^th^ meeting in October 2013. The IMB agreed: that the Polio in the Horn of Africa needs to be treated as a public health emergency with commensurate high-level political commitment, unambiguous and coordinated program leadership, plentiful support to the affected countries, and thoroughness of action. The IMB equally noted that the organisational structure of partner agencies is impeding a coordinated approach. The HOA coordination office thus created, was saddled with the coordination and standardising information sharing, external outbreak response assessment, synchronised SIA planning, support to laboratory activities and progress monitoring. The HOA coordination included partners like Bill and Melinda Gates Foundation (BMGF), US Centers for Disease Control and Prevention, Core Group, United Nations High Commission for Refugees (UNHCR), United Nations Children’s fund Eastern and Southern Africa Regional office (UNICEF ESARO), American Red Cross and United States Agency for International Development (USAID). The coordination by the Kenya public health department at the ministry and Horn of Africa polio partners with the cross-border activity was adjudged by the inter-agency assessors and TAG to be good and have impacted on the outcome.

The Kenya outbreak response was not closed without challenges. There were security issues in the high risk and outbreak zones along the Kenya and Somalia border in Garissa, Wajir, Mandera and in other Counties of West Pokot, Turkana and Samburu. Safety could not be guaranteed coupled with the eventual flight of health workers from the Counties mentioned resulting in staff shortage and high turnover rate in the outbreak areas. The Westgate Mall terrorist attack in Nairobi led to the postponement of external assessment activity and TAG meetings. Despite these security issues, the indigenous team, some of the international and surge staff were not deterred but remained to continue the response activities^23^.

While the donor community provided funding for bulk activities, domestic funding was difficult. Occasionally, there were delays in funds disbursement, late procurement of oral Polio vaccines and inadequate budget line for immunisation and surveillance activities. There were difficulties also in coordination as roles and responsibilities between levels of government were not defined thereby hampering some response activities. Locating and reaching the nomadic and pastoral communities who were always on the move irrespective of the prevailing circumstance was difficult. Tracking and mapping of the nomadic pastoralist and water points were used to reach these populations.

Due to repeated SIAs, program fatigue set in and sustaining the sense of emergency in the face of other competing priorities like the cholera outbreak among others became a major challenge.

Moreover, the ACSM groups had to deal with the controversy from the Catholic Church over the safety of polio vaccine which led to the postponement of 2015; April and May polio campaigns to August. The anti-vaccine sentiments insisted vaccines were unsafe and must be tested ‘before, during and after campaigns’ and called for Polio SIA boycott and withdrawal of support (i.e., for vaccine storage facility, vehicles, and health workers) for SIA implementation in the areas where the Catholic church supports health services. Continuous dialogue including testing of the vaccine was used to allay the anxiety of the anti-vaccine sentiments.

During the first quarter of 2015 (the last phase of the outbreak), the outbreak response witnessed a declining AFP Surveillance performance. The surveillance reviews apportioned solutions to the gaps militating against effective surveillance activities.

Despite these challenges, Kenya stopped the 2013 polio outbreak transmission in the 13^th^ week employing both innovative and traditional methods resulting in the eventual declaration of its closure by the external assessment team, June 2015.

## Limitation Of The Study

Considering the number of years that has elapsed without a Wild poliovirus outbreak in Kenya and given the cross-border migration that exist, the extent to which the pilot, ‘all age immunization activities held from July to August 2013’, in Garissa and Wajir Counties had aided the sustenance of Polio population immunity in the area was not part of this study.

Further, given the migration pattern and insecurity situation in some of the outbreak districts, data received from these areas may have suffered from some form of collection bias. This is, at the back heel that some indigenous independent monitors may not have gone to some areas assigned, alleged to be security prone during data collection. This inability may have confounded the quality of data analyzed and some of the outcomes.

## Figures and Tables

**Table 1:** Kenya AFP Surveillance Indicators, 2012-2015 (Source, HOA Outbreak Response Assessment, June 2015)^24^

Surveillance indicators	2012	2013	2014	2015 (wk 22)
NP-AFP >=2	4.02	3.41	4.07	2.50
Stool adequacy (%) >=80	93.0	80.0	88.0	89.0
Investigation =<2 days of notification (%) >=80	94.0	85.2	85.4	82.0
Specimen arriving Lab =<3days since collection (%) >=80	92.0	85.3	87.3	84.6
Specimen arriving Lab in good condition (%) >=90	99.0	99.8	99.3	100
Lab result at program within 14 days of receipt (%) >=80	94.0	74.0	87.3	81.0
Non-polio enterovirus rate (%) >=10	11.8	13.4	9.0	6.5

**Table 2:** Showing Kenya 2013 wild poliovirus count and time interval in weeks between the first and last case (Source: MoH and WHO Database, 2014)

Country of outbreak	Kenya
Province/County of Outbreak	North Eastern/Garissa
Date of onset first Case	30th Apr, 2013
Date of onset Last Case	14th July 2013
WPV Case Count in 2013	14
WPV Case Count in 2014	0
Time Interval between 1st and last case	Within 13 weeks

## List of Abbreviations

ACSM: Advocacy communication and Social Mobilization
AFP: Acute Flaccid Paralysis
BMGF: Bill and Melinda Gates Foundation
CDC: Center for Disease Control
cVDPVs: Circulating Vaccine-Derived Polioviruses
ESARO: Eastern and Southern Africa Regional office
EPI: Expanded Program on Immunization
GPEI: Global Polio Eradication Initiative
GPLN: Global Polio Laboratory Network
IM: Independent Monitoring
IMB: Independent Monitoring Board
IPC: Interpersonal Communication
IPV: Inactivated Polio Vaccine
KEMRI: Kenya Medical Research Institute
LQAS: Lots Quality Assurance Survey
NIDs: National Immunization Days
OPV: Oral Polio Vaccine
P/TVPs: Permanent/Transit Vaccination Points
SIA: Supplementary Immunization Activities
SIADs: Short Interval Additional Doses
SOP: Standard Operating Procedure
STOP: Stop Transmission of Polio
TAG: Technical Advisory Group
UNHCR: United Nations High Commission for Refugees
UNICEF: United Nations Children’s Fund
USAID: United States Agency for International Development
VDPV: Vaccine-Derived Poliovirus
WHA: World Health Assembly
WHO: World Health Organization
WPV: Wild Poliovirus
